# In Vitro Evaluation of Lithium Disilicate Endocrowns and Post and Core Crowns—A Systematic Review

**DOI:** 10.3390/jfb14050276

**Published:** 2023-05-14

**Authors:** Zeeshan Qamar, Ahmed Mohammed Saad Alghamdi, Naji Khaled Bin Haydarah, Abdulateef Ahmed Balateef, Ahmed Aydhah Alamoudi, Munther Amer Abumismar, Ankita Mathur, Giuseppe Minervini

**Affiliations:** 1 Department of O&MFS and Diagnostic Sciences, Faculty of Dentistry, Riyadh Elm University, Riyadh 13244, Saudi Arabia; 2 Al-Iman General Hospital, Riyadh 12684, Saudi Arabia; 3 Al Dar Albaidhaa 2 PHC, Riyadh 12684, Saudi Arabia; 4 STAT SENSE, Srushti 10, Sector 1 D, Amba Township Pvt. Ltd., Trimandir, Adalaj 382421, Gujarat, India; 5 Department of Periodontology, Dr. D.Y. Patil Dental College and Hospital, Dr. D.Y. Patil Vidyapeeth, Pune 411018, Maharashtra, India; 6 Multidisciplinary Department of Medical-Surgical and Dental Specialties, University of Campania Luigi Vanvitelli, 80138 Naples, Italy

**Keywords:** lithium disilicate, endocrowns, post-and-core, physical properties, dental materials, dentistry

## Abstract

The aim of this systematic review was to summarize the results of the studies that have compared the physical and mechanical properties of lithium disilicate (LDS) endocrowns constructed for posterior teeth to those retained by post-and-core retention systems. The review was conducted following the PRISMA guidelines. The electronic search process was conducted on PubMed-Medline, Scopus, Embase and ISI Web of Knowledge (WoS) from the earliest available date till 31 January 2023. Additionally, the studies were assessed for their overall quality and risk of bias using the Quality Assessment Tool For In Vitro Studies (the QUIN). The initial search resulted in 291 articles, out of which, only 10 studies met the eligibility criteria. In all studies LDS endocrowns were compared with various kinds of endodontic posts and crowns made from other materials. There were no definite pattern or trends observed in the fracture strengths of tested specimens. There was no predilection observed in failure patters among the experimental specimens. No predilection was observed in the fracture strengths of LDS endocrowns when compared to post-and-core crowns. Furthermore, no differences in failure patterns could be observed when both types of restorations were compared. The authors propose standardized testing of endocrowns against post-and-core crowns in future studies. In conclusion, long-term clinical trials are advocated to compare the survival, failure and complication rates of LDS endocrowns and post-and-core restorations.

## 1. Introduction

There are several options to restore extensively damaged or decayed teeth [1]. Conventionally, crowns made from ceramic (usually porcelain) or porcelain-fused-to-metal (PRF) crowns are constructed and cemented or bonded on to prepared crowns. For endodontically treated crowns, a post-and-core restoration is fabricated to improve the retention of the restoration [2]. Several types of endodontic posts have been used. Pre-fabricated post-and-core, customized cast, all-ceramic and fiber-reinforced posts are some types of endodontic retention systems that have been used [3]. The main advantage of a post-retained restoration is the increased retention [4]. However, preparing the tooth for a post weakens the tooth structure and increases the probability of vertical root fractures [5]. These fractures are usually not reparable, and the only usual choice left for their management is usually extraction of the tooth [6].

More recently, endocrowns have been advocated as alternatives for post-and-core crowns [7]. Endocrowns take advantage of adhesives and monolithic ceramic, composite or other materials. An endocrown is a one-piece restoration which requires minimal preparation of the root canal [8]. As opposed to conventional posts (which requires extensive removal of gutta percha and dentine along with extension into the root canal), the endocrown extends just 2–3 mm in to the pulp chamber but not in to the root canal [9]. Nevertheless, endocorowns have some limitations as well. Their use in the anterior or premolar region has not been seen as much as in the molar region [7]. Furthermore, tooth preparations for these crowns are usually technique-sensitive and produced by specialists. Higher costs may limit their use as well. Endocrowns are usually constructed via computer-aid-design and computer-aided-manufacture (CAD-CAM) systems, giving them an additional advantage of being able to be produced ‘chairside’ [10]. Reduction for endocrowns usually require a 2 mm occlusal reduction and and 3 mm axial depth in to the pulp chamber [7]. The gingival margin usually has a butt-joint design. Although several materials such as resin composites and zirconia have been used to construct endocrowns, lithium disilicate (LDS) is a more recent material introduced for their manufacture [10]. In addition to possessing superior flexural and fracture resistance along with excellent esthetic properties, they are able to be milled in to endocrowns [11]. Some studies suggest that LDS has fracture resistance and favours favourable (reparable) fractures compared to post-and-core [12] while others have found no difference. Although prior systematic reviews have been published on endocrowns, none of them focused specifically on LDS endocrowns. Therefore, the aim of this systematic review is to summarize the results of the studies that have compared the physical and mechanical properties of LDS endocrowns constructed for posterior teeth to those retained by post-and-core retention systems.

## 2. Materials and Methods

### 2.1. Focused Question

Using the Participants, Intervention, Control and Outcomes principal described in the Preferred Reporting Items for Systematic Reviews and Meta-analyses (PRISMA) guidelines [13], a focused question was constructed. The question was ‘In patients who need prosthodontic replacement of premolars and molars (participants), are the mechanical and physical properties (outcomes) of LDS endocrowns (intervention) better or worse than (or comparable) to post-and-core-crowns)?’.

### 2.2. Search Strategy

An exhaustive literature search was conducted for mechanical and physical properties of LDS endocrowns. Online electronic databases such as PubMed-MEDLINE, Embase, Scopus and ISI Web of Knowledge (WoS) were explored from the earliest available date till 31 January 2023 without restriction on language. Additional sources like Google Scholar, unpublished studies, conference proceedings and cross-references were explored. Contact with authors was done for any unpublished studies. 

Medical subject headings (MeSH) terms, keywords and other free terms combined with Boolean operators (OR, AND) were used for searching articles. Identical keywords were used for all search platforms following the syntax rules of each database. Databases were searched using the following Medical Subject Headings (MeSH) terms and keywords, [((molar) OR (molars) OR (premolars) OR (posterior teeth)) AND (lithium disilicate) AND (endocrown) AND ((endodontic post) OR (post) OR (post-and-core) OR (post and core)) AND ((strength) OR (fracture) OR (fracture strength) OR (fracture resistance) OR (fatigue) OR (failure) OR (mechanical strength))]. The search strategy was tailored for each database [Table 1]. The search results were downloaded to a bibliographic database to facilitate duplicate removal. The entire search process was carried out by two investigators (AM and GM) independently. Any disagreements were solved by discussion. If a conflict persisted, the judgement of a third reviewer (ZQ) was considered decisive. 

Initially, the titles of the articles in the primary search were read for eligibility. Any irrelevant articles or duplicates were excluded. After exclusion of these articles, the abstracts of the remaining items were read for eligibility to further exclude ineligible articles. Full texts of potentially eligible articles were downloaded and read comprehensively. Furthermore, reference lists of these articles were also read to further find further articles eligible for inclusion. The level of agreement between the two reviewers, calculated by Cohen’s kappa (k), was 0.92 for titles and abstracts and 0.90 for full texts. 

### 2.3. Inclusion Criteria 

It was decided to include the following studies: (1) Prospective clinical studies, (2) Laboratory studies and (3) Studies that focused on the comparison of mechanical and physical properties of LDS endocrowns with those of post-and-core crowns.

### 2.4. Exclusion Criteria

Letters to the editors, commentaries, finite-element analysis studies and case reports were excluded. Studies not in English were excluded via application of language filter. Those papers that fulfilled all selection criteria were processed for data extraction. 

### 2.5. Data Extraction

Using pre-calibrated data extraction forms utilising Microsoft Excel software (version 2303, Microsoft, Redmond, WA, USA), general information corresponding to the following criteria were extracted from the included studies: author/year of publication, type(s) of teeth restored/population details, experimental groups, materials used and tooth preparation/post details. The following outcomes data were extracted: Fracture resistance measurements, marginal adaptation outcomes, failure patterns and overall qualitative outcomes. The data extraction was carried out by two investigators (AM and GM) independently. The data extraction was overseen by a subject matter expert (SME) and any disagreements were solved by discussion with the of third reviewer. (ZQ)

### 2.6. Quality Assessment

The risk of bias in the included studies was assessed using the Quality Assessment Tool For In Vitro Studies (the QUIN) developed by Sheth et al. [14]. Briefly, the following criteria were critically assessed in each study: the aims and objectives, sampling technique, comparison group details, detailed explanation of methodology, operator details, randomization details, outcome measurement, outcome assessor details, blinding and statistical analysis. Overall quality assessments were given to each study corresponding to the range of scores received: high (1–4), medium (5–8) and low (9–12).

The systematic review was registered with the International Prospective Register of Systematic Reviews on 10 November 2022, which was in accordance with the guidelines. (Registration Number CRD42023390072).

## 3. Results

### 3.1. Literature Search

**Figure 1:**
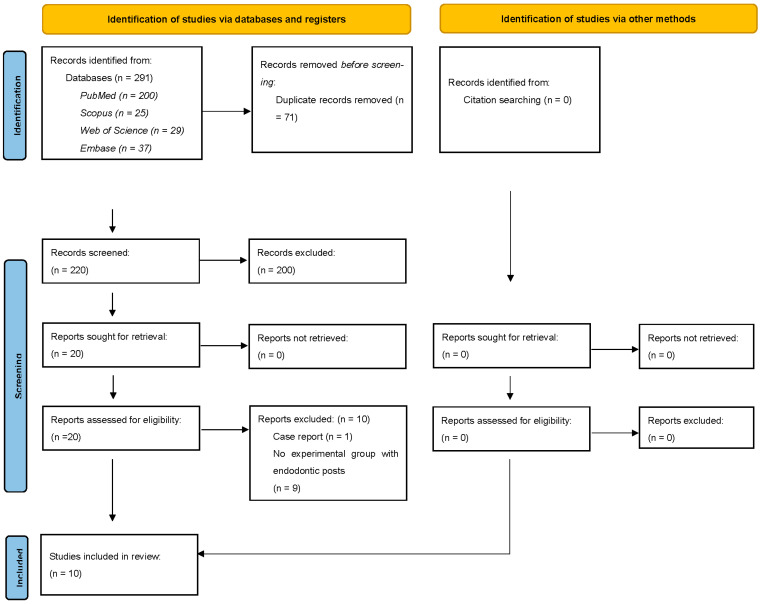
PRISMA 2020 flow diagram for this review.

The literature search process is illustrated in Figure 1. The primary search resulted in 291 items. After removal of 71 duplicate items, titles and abstracts of 220 studies were read to exclude further ineligible studies. At this point, 200 studies were further excluded; therefore, 20 studies were selected for full-text retrieval. Of these, one study was excluded because it was a case report [15] and nine studies were excluded because they did not have an experimental group with endodontic posts [10,16,17,18,19,20,21,22,23]. Therefore, 10 studies were eventually selected for inclusion in this review [11,12,24,25,26,27,28,29,30,31] No studies were found within the reference lists of the selected articles. 

### 3.2. General Characteristics of the Studies

All studies included were in vitro studies [11,12,24,25,26,27,28,29,30,31]. The number of teeth ranged between 30 and 105 [11,12,24,25,26,27,28,29,30,31]. In all studies, LDS endocrowns were compared with various kinds of endodontic posts and crowns made from other materials [11,12,24,25,26,27,28,29,30,31]. In all studies, fracture resistance was measured as load to failure (Newtons) [11,12,24,25,26,27,28,29,30,31]. In two studies, differences were evaluated in margins adaptation [24,26]. In nine studies, failure modes were also recorded [11,12,24,25,27,28,29,30,31]. The detailed experimental groups and controls are presented, along with other characteristics of studies, in Table 2.

### 3.3. Fracture Strength Outcomes

There were no definite patterns or trends observed in the fracture strengths of tested specimens. In the study by Forberger & Gohring, no significant difference was observed between the fracture strengths of endocrowns and post-and-core crowns [24]. In another study, the fracture resistance of endocrowns and post-and-core crowns were similar but still lower than that of unprepared teeth [25]. Lise et al. observed that all specimens survived after 120,000 chewing cycle simulations and there was no difference between endocrowns and post-and-core crowns in terms of fracture strength [12]. In another study, no significant difference was observed between the fracture strengths of endocrowns and post-and-crowns, but all of them performed better than overlay crowns [26]. In the study by Ghoul et al., LDS endocrowns had similar fracture strength to nanoceramic-reinforced endocrowns, zirconia reinforced endocrowns and LDS endocrowns, but all had a higher fracture strength than post-and-core crowns [27]. In contrast, in one study, LDS with a resin composite core (without post) had the highest fracture strength when compared to LDS with a post and LDS endocrowns [28]. In one study, LDS endocrowns had higher oblique fracture strength when compared to post-and-core crowns (with and without ferrule) [29]. When compared to bulk-fill endocrowns, endocrowns were found to have a lower fracture strength [30]. When compared to post-retained zirconia crowns and resin composite endocrowns, LDS endocrowns were found to have a lower fracture strength [31]. Finally, in one study, post-retained crowns, zirconia or LDS endocrowns with 1.5 circumferential ferrule and zirconia crowns with flat occlusal table had comparable higher fracture strengths, but a buccal ferrule resulted in compromised fracture strength [11].

### 3.4. Failure Patterns

There was no predilection observed in failure patterns among the experimental specimens. In five studies, there were equal numbers of root fractures among endocrowns and post-retained crowns [11,12,24,25,29]. When compared to endocrowns and post-and-core crowns, intact teeth were found to have more reparable fractures [25,30]. In two other studies, a higher number of irreparable fractures were observed in endocrowns than in post-and-core crowns [27,28]. In another study, both LDS endocrowns and LDS post-and-crowns had lower numbers of irreparable fractures than did Zr crowns of either design [31]. 

### 3.5. Marginal Adaptation

Marginal adaptation was measured in just two studies. In one study, post-and-core crowns had higher marginal adaption than endocrowns [24]. In the other study, no difference was observed between the two types of prostheses [26]. 

### 3.6. Results of Bias Assessment

All studies adequately described their aims and objectives, comparison groups, outcome measurements, statistics and results [11,12,24,25,26,27,28,29,30,31]. However, none of the studies provided sampling technique, sample size calculation, outcome assessor details or operator details [11,12,24,25,26,27,28,29,30,31]. Randomization was not described in one study [24], but this was the only study in which the investigators were blinded [24]. Nine studies were deemed having a high quality [11,12,24,25,26,28,29,30,31] and one study was graded as medium [27] [Table 3].

## 4. Discussion

The results from studies included failed to draw a conclusive comparison between the mechanical properties of lithium disilicate endocrowns and conventional post-and-core restorations [11,12,24,25,26,27,28,29,30,31]. Nevertheless, the results suggest that LDS endocrowns have similar fracture strength and failure modes to those of post-and-core crowns in majority of the studies [11,12,24,25,26,27,28,29,30,31]. The authors decided to include only molars and premolars in the inclusion criteria of studies because of the similar occlusal forces experienced by them in the posterior region of the dental arch. It is difficult to translate the results of in vitro studies to clinical practice because of several factors that can not be replicated in the laboratory. These include parafunctional habits, oral hygiene, dental practitioner skills and saliva [32,33,34]. Nevertheless, even after undergoing 120,000 chewing cycles, no appreciable differences have been found between the fracture strengths of LDS endocrowns and post-and-core crowns [12].

Marginal adaptation, defined as the distance between the finish line and the restoration margin, is considered one of the major criteria affecting the long term prognosis of ceramic restorations [35]. If a significant marginal gap is present between the tooth and the restoration, luting material will be exposed to the oral environment resulting in its dissolution and consequent microleakage, leading to inflammation of the periodontal tissues, secondary caries and eventually prosthesis failure [35,36,37,38,39]. The assessment of the marginal adaptation of restorations is usually performed using either invasive techniques such as cross sectioning and impression replica or noninvasive techniques such as direct-viewing [39].

Although to date, no comparative clinical trials have been carried out to compare the survival rate of LDS endocrowns and conventional post-and-core crowns, a retrospective study found the 10-year survival rate of predominantly LDS endocrowns to be as high as 99% [18]. A prior systematic review attempted to gauge the performance of endocrowns [40]. However, the authors of that review did not evaluate the material, performance, survival and mechanical properties of LDS endocrowns relative to post-and-core restorations. Before advocating a preference of endocrowns over post-and-core crowns, it is pertinent to synthesize evidence regarding their comparative long-term clinical survival rates, fracture strength and mode of failure. For reparability, it would be preferable for crowns to fail without damaging the tooth structure, particularly avoiding vertical root fractures [41]. In the studies reviewed in the current review, a majority of the studies did not find a significant difference in failure patterns among endocrowns and post-and-core crowns [11,12,24,25,29,31]. To date, very few studies have been carried out to ascertain the impact of pulp chamber extension on the survival or strength of endocrowns [42]. However, a general consensus is that the pulp chamber extension should not exceed the pulpal floor [34]. To date, no studies have attempted to investigate the impact of variability of tooth or pulp shape on the survival of endocrowns. In addition, teeth with deep subgingival margins or root caries may not be suitable for endocrown placement. Nevertheless, this parameter has not been explored significantly in LDS crowns in comparison to other materials. Furthermore, variables such as flexural [43] and compressive strengths [44] have only been partially explored. Finally, no clinical or laboratory studies have attempted to investigate the impact of actual or simulated bruxism and other parafunctional habits. Therefore, future studies should investigate the impact these variables on the performance of LDS endocrowns.

The current review has some limitations. Firstly, due to the heterogeneity of the methodology, results and outcome measurements, a meta-analysis could not be carried out. Therefore, it is difficult to infer any meaningful conclusions regarding the relative properties of the included groups in the studies. Secondly, due to the language restrictions of the investigators, studies only in English were reviewed, further limiting the scope of this review. There were several potential sources of bias detected within the studies. The biggest concerns would be the lack of blinding in majority of the studies which could have influenced the outcomes of the studies. Therefore, the authors propose standardized testing of endocrowns against post-and-core crowns in future studies. Furthermore, long-term clinical trials are advocated the comparison of the survival, failure and complication rates between LDS endocrowns and post-and-core restorations.

## 5. Conclusions

Within the limitations of this review, no predilection was observed in the fracture strengths of LDS endocrowns when compared to post-and-core crowns. Furthermore, no differences in failure patterns could be observed when both types of restorations were compared. Long-term clinical trials are required before LDS endocrowns can be deemed superior to post-and-core restorations.

## Figures and Tables

**Table 1:** A: Keywords for PubMed and Scopus. B: Keywords for Embase and Web of Science.

Domain	Keywords
posterior teeth	(Bicuspids) OR (Premolar) OR (Premolars) OR (Molars, Third) OR (Third Molar *) OR (Tooth, Wisdom) OR (Wisdom Tooth) OR (Teeth, Wisdom) OR (Wisdom Teeth) OR (posterior tooth) OR (teeth, posterior) OR (molar *, first) OR (molar *, second)
Lithium disilicate	(lithia disilicate) OR (lithium Disilicate)
endocrowns	(endocrown *)
post and core	(Post-Core Technic *) OR (Technic *, Post-Core) OR (Post and Core Technic *) OR (Post Technique *) OR (Technique *, Post) OR (Post Technic *) OR (Technic *, Post) OR (Dental Dowel *) OR (Dowel *, Dental)
physical properties	(Propert *, Surface) OR (Surface Propert *) OR (mechanical, properties) OR (Mechanical Phenomenon) OR (Phenomena, Mechanical) OR (Mechanical Concepts) OR (Concept, Mechanical) OR (Processes, Mechanical) OR (Mechanical Process) OR (Flexural Strength *) OR (Resistance *, Flexural) OR (Bend Strengths) OR (Propert *, Flexural) OR (Strength *, Fracture)
**Domain**	**Keywords**
posterior teeth	dens molaris’ OR ‘dentes molares’ OR ‘molar’ OR ‘tooth, molar’ OR ‘molar tooth’ OR ‘bicuspid’ OR ‘bicuspids’ OR ‘dens premolaris’ OR ‘dentes premolares’ OR ‘pre-molar’ OR ‘pre-molars’ OR ‘premolar’ OR ‘premolar teeth’ OR ‘premolars’ OR ‘tooth, premolar’ OR ‘premolar tooth’
Lithium disilicate	lithia disilicate’ OR ‘lithium Disilicate’
endocrowns	ceramic dental crown’ OR ‘ceramic tooth crown’ OR ‘clinical tooth crown’ OR ‘corona clinica’ OR ‘corona dentis’ OR ‘crown’ OR ‘crown work, pulp’ OR ‘crown, dental’ OR ‘crown, tooth’ OR ‘crowns’ OR ‘crownwork, pulp’ OR ‘dental crown’ OR ‘dental crown, ceramic’ OR ‘dental crown, metal’ OR ‘dental crown, metal/polymer’ OR ‘dental crowns’ OR ‘dental prosthetic crown’ OR ‘permanent preformed dental crown’ OR ‘post and core technique’ OR ‘preformed dental crown, permanent’ OR ‘pulp crown work’ OR ‘pulp crownwork’ OR ‘tooth crown margin’ OR ‘tooth pulp crownwork’ OR ‘tooth crown’
post and core	Post and Core’ OR ‘Dental Dowel *’ OR ‘Dowel *, Dental’
physical properties	tiredness’ OR ‘fatigue’ OR ‘bone fracture, stress’ OR ‘fatigue fracture’ OR ‘fracture, fatigue’ OR ‘fractures, stress’ OR ‘stress bone fracture’ OR ‘stress fractures’ OR ‘stress fracture’ OR

* Indicates wildcard.

**Table 2:** Characteristics of the included studies.

Author/Year of Publication	Type(s) of Teeth Restored/Population Details (*n*)	Groups/Materials Used (*n*)	Tooth Preparation/Post Details	Fracture Resistance	Margin Outcomes	Failure Patterns/Modes	Outcomes
Forberger & Gohring, 2008 [11]	Mandibular premolars (*n* = 48)	No treatment Composite only (*n* = 8) EC (LDS) (*n* = 8) LDS crown + Variolink GFP (*n* = 8) RC + Variolink Zirconia (*n* = 8) Zr crown + variolink cement Gold post + crown (*n* = 8) Gold crown + Ketac Cem	Shoulder: 0.8 mm Axial dentine: 2 mm Posts: GFP: 15 mm; Gold: 15 mm; Zr: 10 mm;	No treatment: 849 ± 94.0 N (control) Composite only: 1031.9 ± 266.7 N Endocrown: 1107.3 ± 217.1 N GFP: 1092 ± 307.8 N Zirconia: 1253.7 ± 226.5 N Gold: 1101.2 ± 182.9 N	Marginal continuity EC: 74% GFP: 94.8%	50% of samples had root fractures, irrespective of groups	No significant difference between fracture strengths of endocrowns vs. post-and-core.
Guo et al. 2016 [24]	Mandibular premolars (*n* = 30)	No treatment (*n* = 10) EC (LDS) (*n* = 10) GFP + LDS crown (*n* = 10)	EC: Depth 5 mm, shoulder 2 mm GFP: Core height 3 mm, ferrule 1.5 mm	No treatment: 997.1 ± 166.3 N EC: 479.1 ± 180.6 N GFP + LDS crown: 510 ± 191 N	NA	Intact teeth had more favourable fractures EC and GFP had more root fractures	Fracture resistance of EC (LDS) and GFP + LDS crowns was lower than that of unprepared teeth (*p* < 0.05). No difference between GFP + LDS and EC (LDS) (*p* > 0.5)
Lise et al. 2017 [12]	Premolars (*n* = 48)	EC (LDS/RC)–2.5 mm depth (*n* = 16) EC (LDS/RC))–5.0 mm depth (*n* = 16) GFP + LDS/RC Crown (*n* = 16) Each group restored with LDS or indirect RC (subgroup) (*n* = 8) All groups subjected to chewing cycles (1,200,000).	EC (2.5 mm): 2.5 mm deep, 1 mm wide margin EC (5.0 mm): 5.0 mm deep, 1 mm wide margin GFP: Post with 1.6 mm diameter and 10 mm length	No numerical values provided.	NA	Predominantly root fractures in all experimental groups	100% survival rate of all specimens after 1,200,000 chewing cycles EC (RC) with 2.5 mm depth had highest fracture/load-to-failure resistance (*p* < 0.05). No difference between GFP and 5.0 mm deep ECs.
Rocca et al. 2017 [25]	Premolars (*n* = 48)	Overlays (*n* = 12) EC + (LDS) (2 mm depth) (*n* = 12) EC (LDS) (4 mm depth) (*n* = 12) GFP + LDS crown (*n* = 12) All specimens subjected to thermocycling	EC (2 mm depth): 2 mm depth EC (4 mm depth): 4 mm depth GFP: 5 mm length, core 3.5 mm depth	No numerical values provided.	No difference between margin outcomes of ECs and post-retained crowns. ECs and post-retained higher than overlays.	NA	No difference between 2 mm-deep ECs, 4 mm-deep ECs and post-and-core (*p* > 0.05) Groups 1–3 performed better than overlay controls.
Ghoul et al. 2019 [26]	Mandibular molars (*n* = 80)	GFP + LDS crown (*n* = 20) EC (LDS) (*n* = 20) EC (Zr-LDS) (*n* = 20) EC (Resin nanoceramic) (*n* = 20) Each group subjected to axial and lateral loading, *n* = 10.	1 mm chamfer, 2 mm ferrule, 2 mm occlusal reduction EC depth: 4 mm	GFP: 1347 ± 185 N (axial), 788 ± 92 N (lateral) EC (LDS): 2914 ± 205 N (axial), 1516 ± 205 N EC (Zr LDS): 2279 ± 290 N (axial), 1074 ± 153 N (lateral) EC (Resin nanoceramic): 2752 ± 242 N (axial), 1210 ± 92 N (lateral)	NA	More irreparable (below CEJ) fractures in ECs.	Resin nanoceramic, LDS and Zr-LDS had significant higher fracture strength than post-and-core (*p* < 0.05). LDS had higher axial fracture strength than Zr-LDS and similar to resin. LDS crowns had the highest lateral fracture strength.
de Kuijper et al. 2019 [27]	Molars (*n* = 105)	Endo access cavity only (control) (*n* = 15) GFC crown (*n* = 15) Direct Microhybrid RC crown (*n* = 15) Direct Microhybrid RC crown + GFP (*n* = 15) RC buildup + LDS crown (*n* = 15) LDS/RC core + GFP (*n* = 15) EC (LDS) (*n* = 15)	1 mm chamfer, 2 m ferrule, occlusal reduction 1.5 mm 5 mm apical GP left intact for post No endocrown depth provided	Control: 1890 ± 774 N GFC: 1823 ± 911 N RC crown: 2192 ± 752 N RC crown + GFP: 1830 ± 590 N LDS: 3217 ± 1052 N LDS + GFP: 2697 ± 993 N EC (LDS): 2425 ± 993 N	NA	Glass-fiber reinforcing resulted in more repairable fractures.	LDS with no post had the highest fracture strength (*p* < 0.05), followed by LDS + GFP and EC.
Rayyan et al. 2019 [28]	Premolars (*n* = 27)	EC (LDS) (*n* = 9) LDS/RC core + GFP (no (ferrule) (*n* = 9) LDS/RC core + GFP (ferrule) (*n* = 9) All groups subjected to thermocycling and oblique compressive loading.	EC: 3 mm depth Post: 5 mm of apical GP left intact for post	EC: 594 ± 5.8 N LDS/RC no ferrule: 458.57 ± 5.26 N LDS/RC (ferrule): 491.13 ± 6.93 N	NA	Root fractures observed predominant in all groups.	Endocrowns had highest oblique fracture strength compared to other groups (*p* < 0.05)
Sedrez-Porto et al. 2019 [29]	Molars (*n* = 63)	Endocrowns LDS Filtek 350 XT RC Filtek 2350 XT RC + Multipurpose adhesive 2350 XT + Universal adhesive Bulkfill (Filtek) Post-retained Filtek 2350 (incremental) Filtek 2350 (bulk-fill)	EC: 2 mm distal root depth, 1 mm depth for other roots	Sound tooth: 2149.9 ± 13.8 N LDS: 1748.5 ± 559.3 N Filtek 2530: 2292.3 ± 716.8 N Filtek + Multipurpose adhesive: 2546.3 ± 216.8 N Filtek 2350 + Universal adhesive: 2583.7 ± 612.2 N Bulkfill: 3363.1 ± 123.9 N Post-retained Filtek 2350: 2451.6 ± 484.5 N Filtek 2350 + Multipurpose adhesive: 2774 ± 578.8 N Bulkfill: 2861.2 ± 424.1 N	NA	Irreprable fractures: Natural teeth: 14% EC repairable fractures: 21% Post-retained: 57–81%	The bulk-filled endocrowns exhibited highest fracture strength compared to all other groups (*p* 0.05).
Hassouneh et al. 2020 [30]	Premolars (*n* = 70)	EC (*n* = 30) RC (*n* = 10) LDS (*n* = 10) Zr (*n* = 10) Post-retained (*n* = 30) RC (*n* = 10) LDS (*n* = 10) Zr (*n* = 10) No treatment (*n* = 10)	EC retention depth: 4 mm Post length: 3 mm projection in the post-build up, 3–5 mm of GP left intact	EC RC: 758.1 ± 105.2 N LDS: 547.4 ± 141.5 N Zr: 460 ± 112 N Post-retained RC: 477 ± 134.4 N LDS: 534.1 ± 119.1 N Zr: 815.6 ± 87.6 N No treatment: 947.4 ± 223 N	NA	Zr crowns had higher % of catastrophic fractures	Post-retained Zr crowns and resin composite ECs exhibited higher fracture strength compared to other materials (*p* < 0.001). Zr crowns had the highest unrepairable fractures (*p* < 0.05).
Ahmed et al. 2022 [31]	Premolars (*n* = 56)	EC (LDS/Zr) Flat occlusal table (no ferrule) (*n* = 16) EC + 1.5 mm circumferential ferrule (*n* = 16) EC + 1.5 mm buccal ferrule (*n* = 16) Post-retained: LDS/Zr crowns + Zr post (*n* = 16)	Ferrule: 1.5 mm (buccal and circumferential) EC retention depth: 2 mm	LDS 661 ± 143 N (buccal ferrule), 870 ± 167 N (no ferrule), 1225 ± 172 N (circumferential ferrule) Zr 1165 ± 172 N (circumferential ferrule), 1391 ± 309 N (no ferrule), 857 ± 136 N (buccal ferrule) Post and core 1440 ± 316 N (LDS and LDS)	NA	Only two post-and-core crown specimens (25%) with a favourable failure mode.	Post-retained crowns, Zr/LDS ECs with 1.5 circumferential ferrule and Zr with flat occlusal table had higher fracture strengths than other groups (*p* < 0.05), with no difference between them (*p* > 0.05).

EC–endocrowns, LDS–Lithium Disilicate, GFP–Green Fluoroscent Protein, RC–Root Canal, Zr–Zirconia, N–Newton, NA–Not Applicable.

**Table 3:** Quality assessment.

Study	Aims & Objectives	Sample Size	Comparison Group	Methodology	Sampling Technique	Operator Details	Randomization	Outcomes Measurement	Outcomes Assessor Details	Blinding	Statistical Analysis	Results	Overall Quality
Forberger & Gohring, 2008 [11]	Yes	No	Yes	Yes	No	No	No	Yes	No	Yes	Yes	Yes	High
Guo et al. 2016 [24]	Yes	No	Yes	Yes	No	No	Yes	Yes	No	No	Yes	Yes	High
Lise et al. 2017 [12]	Yes	No	Yes	Yes	No	No	Yes	Yes	No	No	Yes	Yes	High
Rocca et al. 2017 [25]	Yes	No	Yes	Yes	No	No	Yes	Yes	No	No	Yes	Yes	High
Ghoul et al. 2019 [26]	Yes	No	Yes	Yes	No	No	No	Yes	No	No	Yes	Yes	Medium
de Kuijper et al. 2019 [27]	Yes	No	Yes	Yes	No	No	Yes	Yes	No	No	Yes	Yes	High
Rayyan et al. 2019 [28]	Yes	No	Yes	Yes	No	No	Yes	Yes	No	No	Yes	Yes	High
Sedrez-Porto et al. 2019 [29]	Yes	No	Yes	Yes	No	No	Yes	Yes	No	No	Yes	Yes	High
Hassouneh et al. 2020 [30]	Yes	No	Yes	Yes	No	No	Yes	Yes	No	No	Yes	Yes	High
Ahmed et al. 2022 [31]	Yes	No	Yes	Yes	No	No	Yes	Yes	No	No	Yes	Yes	High

## Data Availability

Not applicable.
